# Integrative Modeling of Small Artery Structure and Function Uncovers Critical Parameters for Diameter Regulation

**DOI:** 10.1371/journal.pone.0086901

**Published:** 2014-01-31

**Authors:** Ed VanBavel, Bilge Guvenc Tuna

**Affiliations:** Department of Biomedical Engineering and Physics, Academic Medical Center, Amsterdam, The Netherlands; University of Arizona, United States of America

## Abstract

Organ perfusion is regulated by vasoactivity and structural adaptation of small arteries and arterioles. These resistance vessels are sensitive to pressure, flow and a range of vasoactive stimuli. Several strongly interacting control loops exist. As an example, the myogenic response to a change of pressure influences the endothelial shear stress, thereby altering the contribution of shear-dependent dilation to the vascular tone. In addition, acute responses change the stimulus for structural adaptation and vice versa. Such control loops are able to maintain resistance vessels in a functional and stable state, characterized by regulated wall stress, shear stress, matched active and passive biomechanics and presence of vascular reserve. In this modeling study, four adaptation processes are identified that together with biomechanical properties effectuate such integrated regulation: control of tone, smooth muscle cell length adaptation, eutrophic matrix rearrangement and trophic responses. Their combined action maintains arteries in their optimal state, ready to cope with new challenges, allowing continuous long-term vasoregulation. The exclusion of any of these processes results in a poorly regulated state and in some cases instability of vascular structure.

## Introduction

Local blood flow is matched to metabolic needs by tight regulation of the diameter of small arteries and arterioles. The regulation of resistance vessel diameter includes both acute control of smooth muscle cell (SMC) contractile activity and, on a longer time scale, adaptation of vascular wall structure [1]. This control system is crucial for continuous adaptation to changing metabolic needs, normal development and adaptation to e.g. regular exercise [2] and pregnancy [3]. Control of resistance artery caliber is affected in various cardiovascular pathologies. As an example, increased vascular resistance is found in established hypertensive disorders. This is a structural change, characterized by eutrophic inward remodeling, i.e. without a loss or gain of wall cross-sectional area [4]. Such eutrophic inward remodeling reflects the rearrangement of existing wall material around a smaller diameter [1]. Hypertrophic outward remodeling is observed under high flow [5], and a flow effect contributes to collateral vessel outgrowth in the presence of stenosis of one of the major coronaries[6].

Over the last years it is becoming increasingly clear that vascular adaptation involves a continuum of processes acting in strongly diverging time domains [1], [7], ranging from changes in SMC tone at the seconds to minutes scale to trophic responses in days to weeks. Intermediate processes include the reorganization of the existing vascular cells and matrix [8]. These processes are likely to interact for several reasons. Firstly, they share stimuli. Higher blood pressure elevates wall stress, which is believed to be a stimulus for both the acute myogenic response and vascular growth [9]. Similarly, higher flow induces both acute shear-induced dilation [10] and slower outward remodeling [5], [11]. Secondly, vascular adaptation is a closed-loop process, where responses feed back on the stimuli. The myogenic response upon a change in pressure will cause a partial return of wall stress towards its initial level. Therefore, a possible hypertrophic response to the pressure elevation would depend on the myogenic strength, and vice versa. Experimental data indeed indicate a link between impaired myogenic responsiveness and hypertrophic rather than eutrophic remodeling in hypertension and diabetes [12].

There are many quantitative differences between resistance vessels of varying diameter and from different vascular beds. Yet, all these vessels have evolved into a similar state: both wall stress and shear stress are regulated, by adaptation of inner radius and wall thickness. SMC length may vary, but by far not in proportion to the vascular caliber [1]. Active and passive radius-tension relations are matched [8], with peak active tension occurring at ∼90% of maximal matrix distension. Finally, all resistance vessels maintain an intermediate level of basal tone, providing vascular reserve. This state of the resistance vessels allows adequate and rapid adaptation to changing conditions. However, it is far from clear how such an 'optimal state' is accomplished and maintained, considering the complex interactions that occur at a large range of time scales.

In order to unravel the complex regulation of vascular caliber and wall properties, we integrate the above processes into a simulation model of the resistance artery wall. The model includes biomechanics of a vessel subjected to pressure and flow, and four biological adaptation processes: regulation of tone, maintenance of smooth muscle cell length, organization of the existing matrix structure and hyper/hypotrophy of the vascular wall. The model tests the relevance of these processes, and provides predictions for short-term and chronic effects of pressure, flow, and vasoactive agents. It indicates how these four adaptation processes in interaction with wall mechanics maintain resistance vessels in their optimal state during their adaptation to changing stimuli. The model finally identifies hitherto unstudied parameters that critically determine vascular wall homeostasis.

## Methods

### Model description

Below we provide a description of the model, followed by details on the implementation in Matlab. Extensive supporting information is provided on the experimental data underlying the modeling choices. Model symbols and values for parameters are listed in Tables 1 and 2.

**Table 1:** Definition of the variables in the model.

Variables	Symbol	SI Unit	Description
**state variables**	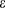	-	matrix strain (dot indicates time derivative)
	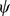	-	tone, 0–1
	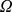	-	SMC span, fraction of circle
	*r_mslack_*	m	Slack midwall radius of matrix
	*wCSA*	m^2^	Wall cross-sectional area
**input variables**	*P*	Nm^2^	Pressure
	*Q*	m^3^s^−1^	Flow
	*CON*	Nm^−2^	extrinsic constrictor, expressed as equipotent stress
	*DIL*	Nm^−2^	extrinsic dilator, expressed as equipotent shear stress
**derived variables**			
*dimensions*	*CSA_j_*	m^2^	CSA of element type *j, j = p(assive), a(ctive) or c(ytoskeleton)*
	*h*	m	wall thickness
	*r_m_*	m	midwall radius
	*l*	m	SMC length
	*r_i_*	m	internal radius
	*r_islack_*	m	slack internal radius
	*r_i100_*	m	internal radius of the passive vessel at 100 mmHg
	*r_iopt_*	m	internal radius at optimal active stress capacity
*stresses*	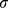	Nm^−2^	mean wall stress
	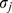	Nm^−2^	stress on element type *j, j = p(assive), a(ctive) or c(ytoskeleton)*
	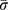	Nm^−2^	stress in mechanical equilibrium, based on Laplace
	*T*	Nm^−1^	wall tension, i.e. force per vessel length
	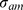	Nm^−2^	capacity for active stress generation
	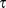	Nm^−2^	wall shear stress
*tone related*	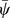	-	equilibrium tone
	*TRF*	-	tone reducing factor

**Table 2:** Definition of parameters and their values in the model.

Parameters	Symbol	SI Unit	Description	Value
Rates	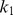	m^2^N^−1^s^−1^	rate constant mechanical radius changes	1×10^−6^
	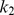	s^−1^	rate constant tone changes	0.005
	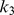	m^−1^s^−1^	rate constant SMC length adaptation	3.75
	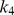	s^−1^	rate constant matrix slack length adaptation	2×10^−5^
	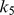	m^2^N^−1^s^−1^	rate constant trophic responses	1×10^−10^
composition	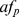	-	fraction of wall area covered by matrix	1/3
	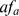	-	fraction covered by active elements	1/3
	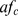	-	fraction covered cytoskeletal elements	1/3
Passive	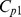	Nm^−2^	constant in passive stress-strain curve	55,000
	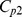	Nm^−2^	constant in passive stress-strain curve	0.0186
	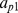	-	constant in passive stress-strain curve	1.5
	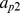	-	constant in passive stress-strain curve	24
Active	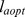	m	optimal SMC length for active stress	106.18×10^−6^
	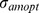	Nm^−2^	maximal active stress at optimal length	250,000
	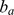	m	width of active length-stress curve	45.868×10^−6^
cytoskeleton	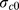	Nm^−2^	cytoskeletal stress at *l*_co_	250,000
	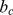	m^−1^	steepness cytoskeletal stress curve	30
	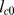	m	length parameter in cytoskeletal stress curve	118.92×10^−6^
Tone	*hc*	-	Hill coefficient for activation curve	3
	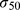	Nm^−2^	stress for half-maximal activation	30,000
	*hd*	-	Hill coefficient for relaxation curve	3
	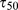	Nm^−2^	shear stress for half-maximal relaxation	5
	*ecf*	-	endothelial cell function	1 in default
SMC adaptation	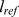	m	reference SMC length for adaptation	80×10^−6^ in default
matrix remod	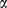	-	effect of tone on inward remodeling	0.65
	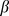	-	effect of strain on outward remodeling	1 in default
Growth	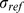	Nm^−2^	reference stress for growth	30,000 in default
miscellaneous	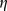	Nsm^−2^	blood viscosity	0.0035

**Figure 1:**
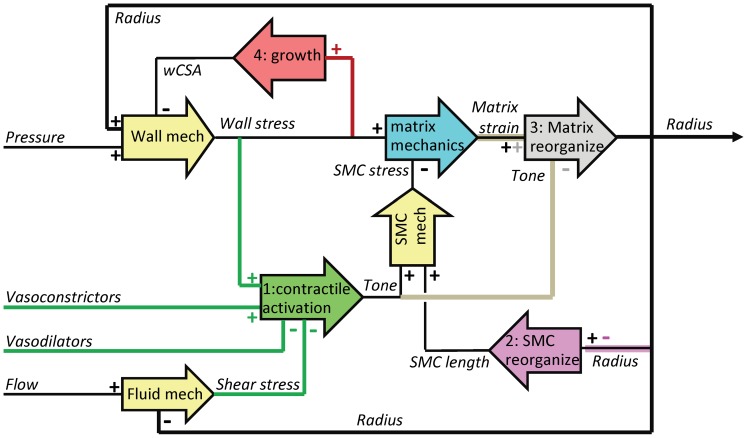
Schematic representation of the model, with color-coded biomechanics and vascular adaptation. The arrow blocks reflect the processes, with input and output signals. For each input, the plus or minus sign indicates a positive or negative effect on the output. Note that a plus sign also indicates a reduction in output upon a reduction in input. Double signs indicate two separate effects of the same input. Yellow blocks are static mechanical relations, other blocks are causal with first order dynamics; outputs of the five causal blocks define the state of this system. Mechanical loading is represented by the static relations (yellow blocks) and by strain following stress with first order kinetics (blue). The four adaptation processes are: Adaptation 1: smooth muscle cell contractile activity, tone (green lines and block). Adaptation 2: SMC reorganization (plasticity, pink). Adaptation 3: eutrophic matrix remodeling (grey). Adaptation 4: wall cross-sectional area growth (red).

Figure 1 illustrates the elements in this model and their relationships. We consider a single resistance artery segment with four input parameters: pressure, flow, vasoconstrictors and vasodilators. Stress-carrying constituents are the matrix and the SMC. Four biological adaptation processes are included that affect biomechanics of matrix and SMC, and therefore the radius: 1) SMC contractile activation, tone, is influenced by several stimuli and inhibitors (green). 2) SMC cells can reorganize their position in the wall, a process referred to as SMC plasticity (pink). 3) Reorganization of the existing matrix, i.e. eutrophic vascular remodeling (grey). 4) A tissue growth response (red). These processes are strongly coupled, in part by mechanical relationships (yellow).

### Mechanics: Vascular wall composition and mechanical loading

**Composition and arrangement.** The three stress-carrying elements are the passive matrix (subscript *p*), active SMC contractile filaments (*a*) and SMC cytoskeleton (*c*). They each cover a fraction (*af*) of the wall cross-sectional area (*wCSA*).
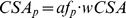
(1)

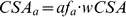
(2)

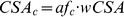
(3)


These elements are arranged in a parallel fashion. Therefore, mean wall stress (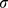) depends on stresses on the individual elements (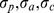) according to:
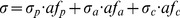
(4)


**Matrix stress.** A non-linear stress-strain relation was defined without further assumptions with respect to the contribution of collagen and elastin. The relation is similar to that of Jacobsen [13], and includes four parameters (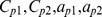):
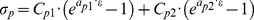
(5)


With strain 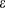
defined as the relative extension of the mid-wall radius (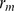) from its slack value (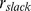):
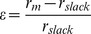
(6)


**Smooth muscle cell active stress.** The capacity for active stress generation (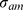) was taken as function of cell length rather than radius in order to accommodate for rearrangement of the SMC during adaptation. 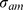 is a bell-shaped function of the SMC length (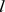), with a maximum of 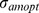 at cell length 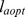. Width of this curve is defined by parameter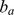.
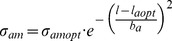
(7)


The actual active stress 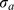 depends on this capacity and the degree of contractile activation. As was done in previous experimental work [14] and modeling studies of others [13], [15], we defined a dimensionless tone (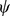) as the ratio of actual and maximal active stresses at the same SMC length, and used this to calculate actual active stress:
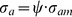
(8)


The factors driving adaptation of tone are indicated below.

**Smooth muscle cell cytoskeletal stress.** Based on the work of Martinez-Lemus *et al*. [16], a cytoskeletal brake was incorporated that prevents stretch of the SMC beyond a given length. The brake was modeled as a very steep dependence of cytoskeletal stress on cell length 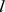, set by parameters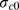 for the stress at 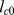 and steepness parameter 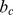.
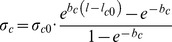
(9)


**Mechanical equilibrium.** The Laplace relation was assumed to hold, with pressure *P*, internal radius *r_i_,* wall thickness *h* and equilibrium wall stress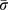:
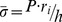
(10)


Changes in geometry, activation or pressure lead to mechanical disequilibrium. Assuming first order kinetics, the strain adapts towards a new equilibrium according to:
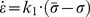
(11)


Internal radius, midwall radius, *wCSA* and wall thickness are related, with any two of these defining the vessel dimensions (relations not shown).

### Adaptation 1: smooth muscle cell contractile activity (tone)

As was done in previous experimental work [14] and modeling studies of others [13], [15], we defined tone as the ratio of actual and maximal active stresses at the same SMC length. A fully relaxed vessel has tone equal to zero; a fully activated one has tone equal to unity (Figure S1 in file S1). We included four factors that are known to influence tone: pressure via the wall stress-induced myogenic response [17] (Figure S2A in file S1), flow via shear stress-dependent dilation (Figure S2B in file S1), and extrinsic constrictors and dilators. Endothelium-independent extrinsic constrictors (CON) added linearly to the effect of wall stress. Their combined effect on tone follows a Hill curve with 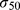 as the stress for half-maximal activation and *hc* as the Hill coefficient. Effects of extrinsic endothelium-dependent dilators (*DIL*) and shear stress (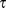) were incorporated by fractional reduction of tone, defining a tone-reducing factor (TRF, between 0 and 1) as previously done by Cornelissen *et al.* [15]. No assumptions were made on the relative contribution of NO, prostaglandins or EDHF's in dilator effects. Endothelium-dependent constriction respectively endothelium-independent dilation were not considered but could be implemented by negative values of *DIL* and CON.

Equilibrium tone (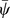 ), when adaptation is complete, thus becomes
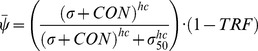
(12)


The effects of shear stress and extrinsic dilators (*DIL)* on reduction of tone were incorporated as a Hill curve with 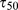 as the stimulus for half-maximal dilation and *hd* as the Hill coefficient. Endothelial cell function (*ecf)* is included for testing the effect of endothelial cell dysfunction, and is unity in default.
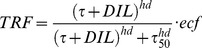
(13)


Shear stress was calculated from the flow *Q*, which was taken as a constant input parameter, using Poiseuille flow and constant fluid viscosity 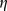:
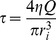
(14)


The rationale for using four drives for tone is that both wall stress and shear stress are subject to intrinsic negative feedback in this model of a single segment, while levels of extrinsic vasoactive agents are not. Both extrinsic constrictors and dilators were expressed in Nm^−2^, and should be interpreted as the equipotent wall stress and shear stress effects of extrinsic and constant vasoactive agents. Actual tone (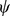) would not instantaneously follow changes in input conditions. We implemented linear first order kinetics for changes in tone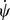, with a rate constant 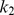:
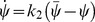
(15)


### Adaptation 2: SMC span and length ( SMC plasticity)

**Figure 2:**
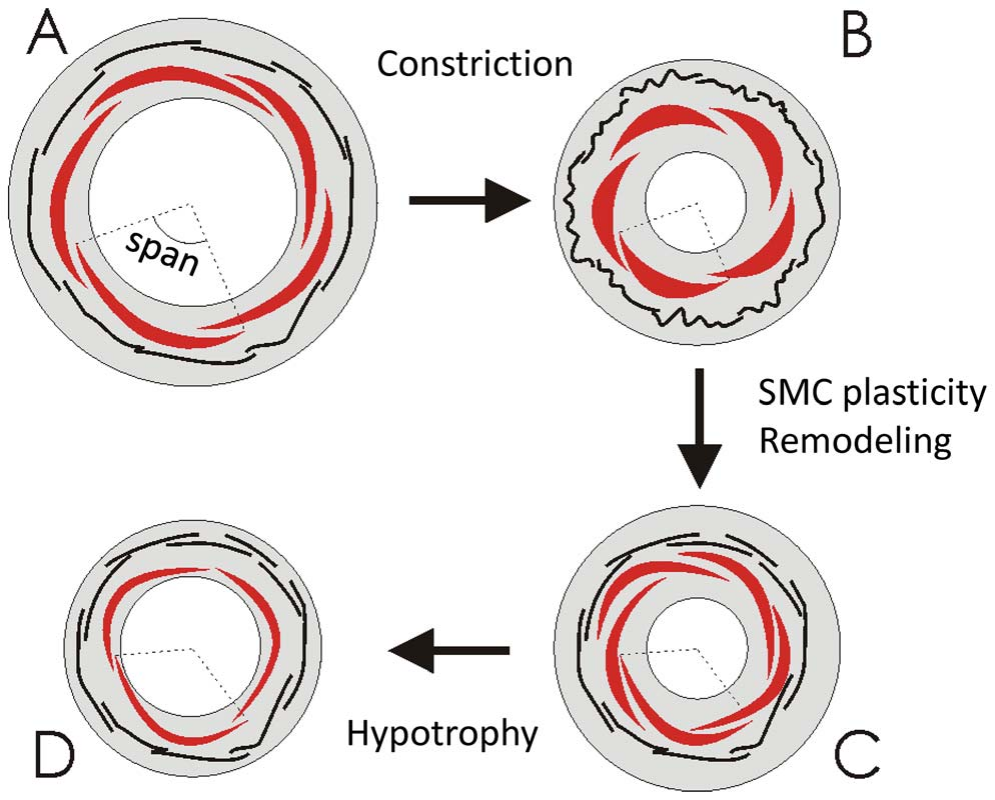
Illustration of vascular adaptation, shown for the effect of a vasoconstrictor. 2A depicts a functional vessel with SMC (red) and matrix elements (black lines). 2B: Following constriction, SMC become shorter but SMC span (dotted lines) remains constant. Matrix becomes slack. 2C: SMC plasticity then normalizes SMC length while increasing the span. Eutrophic matrix remodeling straightens the matrix fibers. 2D: Finally, hypotrophy and hypoplasia occur due to the lower wall stress, resulting in a vessel that has become functional at a structurally smaller radius and thinner wall.

SMC length remains relatively constant when vessels develop from very small arterioles to arteries (Figure S3A in file S1). As a consequence, a single SMC will cover a strongly decreasing part of the wall circumference during such development. We defined the SMC span (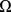) as the part of the vessel circumference that is covered by a single cell (Figure 2A). Evidence suggests that SMC length is regulated at short time scales. Thus, a constriction causes SMC to become shorter, followed by relengthing within hours despite maintained presence of vasoconstrictors and maintained vascular narrowing [18] (S3B in file S1). The cells can only do so by repositioning and increasing their span (Figure 2A-C). The consequence of such adaptation is that active and cytoskeletal stresses for specific radii change. We indeed found that maximal active tension shifts to smaller radii during maintained constriction [19] (Figure S3C in file S1).

In the model we included regulation of SMC length by adaptation of span, i.e. SMC plasticity. SMC were modeled to be arranged perpendicularly to the vessel axis, in accordance with the observation of a low pitch in resistance arteries [20]. Cell span, cell length and vessel radius are related. Ignoring radial dispersion of SMC position, the relation between length, mid-wall radius and span is
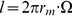
(16)


SMC plasticity was incorporated by an adaptation of cell span that is driven by a deviation of length from its reference value (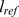), following linear first order kinetics with rate constant 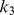.
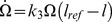
(17)


Including 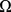 on the right side of equation 17 was based on geometry and ensured that the relative rate of length change as a result of plasticity is proportional to the ratio of active and reference cell length.

### Adaptation 3: eutrophic matrix remodeling

Small arteries are able to remodel the existing matrix elements around a smaller or larger lumen [1] (Figure 2C and Figure S4A in file S1). Inward eutrophic remodeling is also a hallmark of various hypertensive disorders [21] (figure S4B in file S1). After experimental interventions such as flow diversion, acute changes in tone are followed by eutrophic remodeling in the course of a few days [22] (Figure S4C in file S1). In a range of studies on pressurized vessels in organoid culture, we observed that continuous deep tone causes inward remodeling in the course of days. Active constriction rather than only passive collapse was needed for such remodeling. Vasodilation and distension inhibited inward remodeling [23] (S4D in file S1). Coronary resistance vessels demonstrated outward remodeling when vasodilated and strained by pressure *in vitro* [24]. These data show that outward remodeling is induced by long-term strain, while active tone results in inward remodeling. We showed that this tone-remodeling coupling involves transglutaminases, matrix cross-linking enzymes [25]. We incorporated these experimental findings as adaptation of slack radius (i.e. the vasodilated radius at zero load) towards a state where inward remodeling by tone and outward remodeling by matrix strain are in balance:
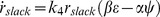
(18)


With 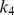 the rate constant for matrix remodeling. Proportionality with 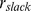 was included for geometric reasons. 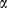 and 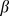 are dimensionless parameters setting the magnitude of tone and strain effects on remodeling.

### Adaptation 4: wall cross-sectional area growth

Larger vessels have thicker walls, and based on the Laplace relation wall stress is relatively constant when considering vessels of quite different caliber [26]. Experimental interventions that increase radius, eventually also cause hypertrophy, normalizing wall stress (Figure S5A in file S1). As an example, chronic changes in flow cause remodeling as well as hypertrophy or hypotrophy at increased respectively decreased flow [27] (Figure S5B in file S1). As a final adaptation process we therefore included a slow trophic response to changes in wall stress, resulting in an increase of the wall cross-sectional area at high wall stress and the reverse (Figure 2D) at low wall stress. The relative area covered by the three stress-carrying components was kept constant during such adaptation.
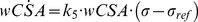
(19)


### Model implementation and simulation

The model response to e.g. a step change in pressure depends on five simultaneous and heavily intertwined processes: progress towards a mechanical balance, and the four listed biological adaptation processes. The model state is defined by vector 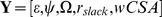. Its evolution in the 5D state space,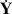, at any time depends on this state and the input 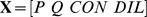 at that moment:
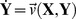
(20)


with 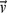
a vector defining the progress in terms of the current state and the input parameters, according to the above relations. Equilibrium points for constant 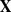 are those points where 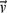 vanishes. This final steady state was equal for a large range of possible initial conditions. In particular, we established that the model progresses to the same steady state when varying initial levels of the state variables between 10% and 1000% of default values, or smaller where geometric (e.g. limited wCSA to maintain r_i_>0) or numerical constrains (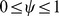) were met (not shown).

We simulated the dynamical behavior of this system, but present mainly steady state behavior in the current results. The default input conditions were constant at *P* = 80 mmHg, *Q* = 0.25 µL•s^−1^, and absence of vasoactive agents. Starting from the equilibrium state of the vessel for these conditions, step changes in these inputs were applied. This was done for various combinations of vascular adaptation. Thus, *MECH* is the model depicting the pure mechanical response in the absence of any adaptation. Accordingly, in *MECH*, we allowed 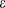 to vary while keeping 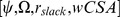 constant at their steady state values under default conditions. This was implemented by setting the rate constants 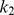 to 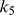 to zero. The functional behavior (*FUNCT*) combines mechanical and tone responses, with 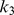 *to* 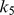 set to zero. *PLAST* also allows SMC plasticity. *REMOD* further includes eutrophic remodeling. *GROWTH* is the full 5-state model with also trophic responses. 'Software knock-out' models *FUNCTKO, PLASTKO and REMODKO* are based on the full model with exclusion of a single adaptation process. In addition to these KO models, we tested the effect of modification of several key parameters in *GROWTH*.

While strain 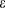 is a state variable in the simulations, the inner radius 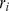 is a physiologically more relevant reflection of the state. The results therefore present the latter. For the same reason, slack inner radius replaces slack midwall radius and cell length replaces cell span in presentation of the data. Simulations were executed in S.I. units, while the more common units were used for presentation of the results.

Simulations were performed in Matlab version 7.9.0.529 (Mathworks, Natick MA, USA). The model includes processes at highly different time scales. Accordingly, a solver for stiff systems was used (Matlab ODE15S). Relative tolerances were set to 10^−5^, absolute tolerances were 10^−5^ for strain, 0.01 µm for slack radius, 0.001 for tone and span, and 1 µm^2^ for wCSA. Time needed for each simulation was around a second, depending on the model and input, using an Intel Core2 Quad processor.

## Results

### Biomechanics under default input conditions

**Figure 3:**
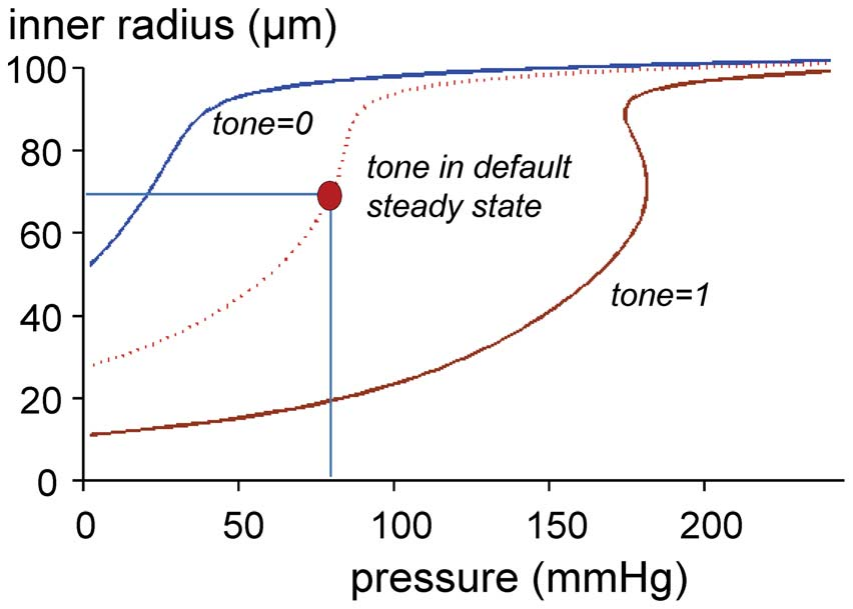
Pressure-radius relation in the absence of adaptation. The dotted line depicts the effect of pressure after steady state under default conditions has been accomplished. This line is characterized by a very steep dependency of radius on pressure around the working point (red circle). Blue and brown lines depict this relation at respectively full relaxation (tone = 0) and full activation (tone = 1).

Starting from equilibrium under default pressure and flow, we ‘froze’ all further adaptation and evaluated the pure mechanical response to changing pressure (the *MECH* model). Additionally, we determined the pressure-radius relations at full relaxation (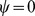) and full activation (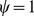). Figure 3 indicates that the mechanical response to increasing pressure is characterized by non-linear distension. Without adaptation, the internal radius at the working point steeply increased at higher pressure, rendering the vessel very sensitive to pressure variations. Figure S1 in file S1 depicts a more extensive biomechanical analysis under pressurized and isometric conditions as well as a comparison with experimental data.

### Effects of vascular adaptation

**Figure 4:**
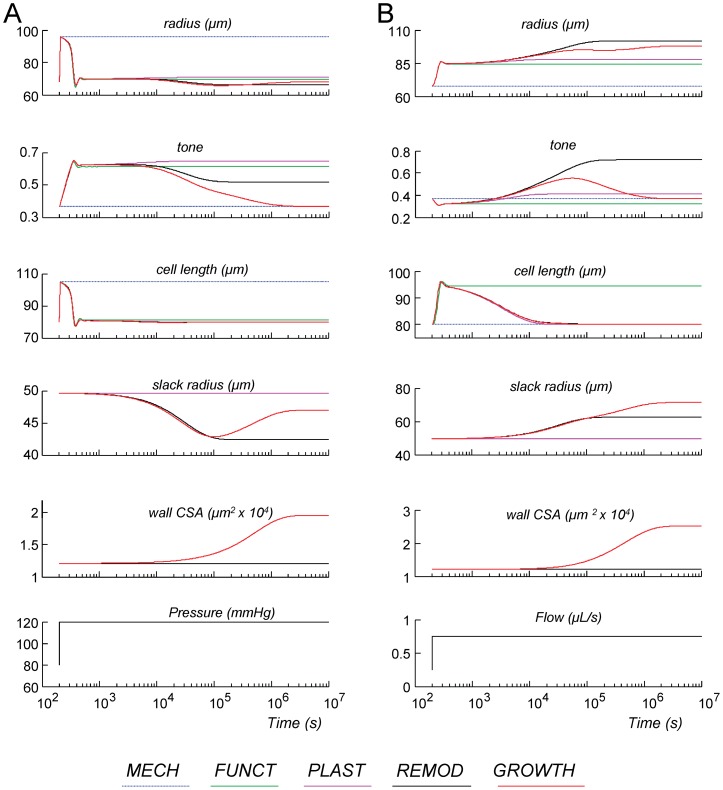
Dynamic responses of the various models to a step in pressure (4A) and flow (4B). Note the logarithmic time axis.

Small artery adaptation is required to ensure that internal radius is insensitive to pressure variations, while larger flow should induce an increase in radius in order to accommodate such flow without undue viscous loss. Figure 4 shows examples of dynamic responses to a step increase in pressure (Figure 4A) and flow (Figure 4B). These responses are indicated for models with increasing complexity, ranging from the mechanical response (*MECH*) to the full model with all adaptations (*GROWTH*).

The progression of the inner radius in Figure 4A suggests that for counteracting the distending effect of pressure, regulation of only tone (in *FUNCT*) may seem sufficient. Yet, this would require a very large and maintained increase in tone. This is energetically less favorable and not supported by data on tone in high pressure versus low pressure arteries. Including regulation of SMC length (added in *PLAST*) did not strongly affect the responses to pressure. Matrix remodeling (added in *REMOD)* contributed to regulation of radius by causing a structurally smaller vessel with less tone as compared to *FUNCT*. This led to substantial eutrophic inward remodeling, as reflected by the lower slack inner radius in Figure 4A. Inclusion of wall hypertrophy (added in *GROWTH)* contributed to regulation by providing a thicker wall while fully restoring both inner radius and tone to their levels before the pressure step.

For a step increase in flow (Figure 4B), in *FUNCT* a small reduction of tone resulted in sufficient vasodilation to normalize shear stress. In *REMOD*, the initially reduced tone and increased radius both induced an increase in slack radius. This eutrophic outward remodeling continued until sufficient myogenic tone developed to balance this process, rendering a very thin-walled vessel with high activation. A hypertrophic response (*GROWTH*) was needed to normalize tone. This hypertrophy was a secondary response, induced by the increased wall stress (Laplace relation) and not initially by the flow, illustrating the complexity of the interactions in such angioadaptation.

**Figure 5:**
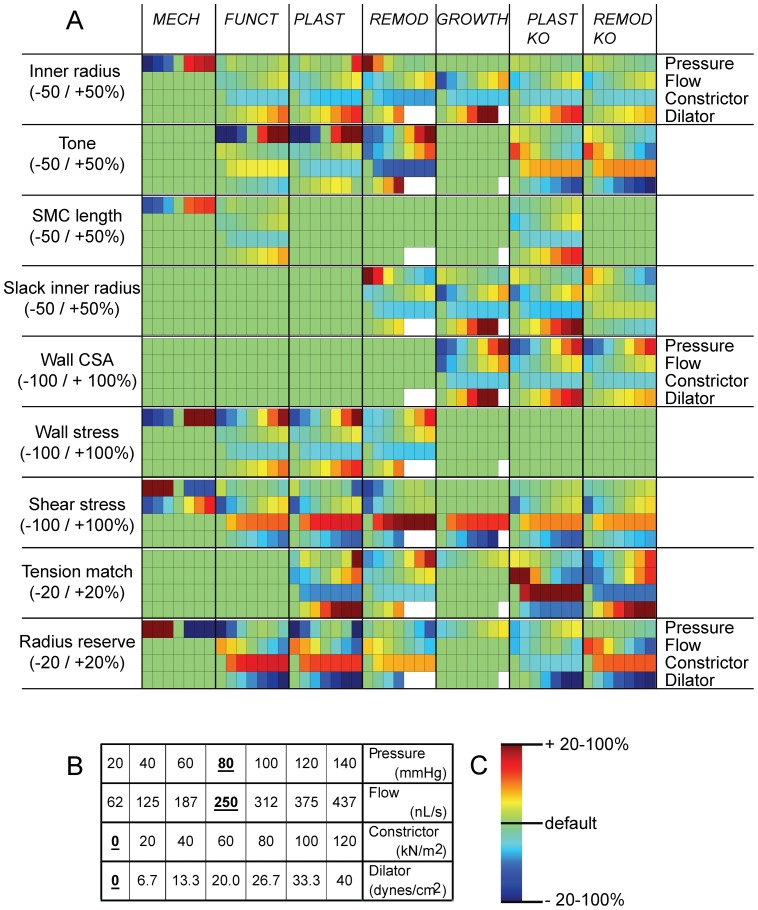
Regulation of the vessel state under varying input. 5A shows the vessel state (inner radius, tone, SMC length, slack inner radius, wall CSA) and additional quantities with pathophysiological significance (wall stress, shear stress, tension match and radius reserve). Each colored block represents a simulation. Shown are deviations from the default results when any of four stimuli is changed over the range indicated in 5B (bold underlines: default values, constrictor and dilator expressed as equivalent wall stress and shear stress, respectively). These results are depicted in a color scale (5C) for the eight considered models. White blocks were unstable.

In order to further investigate whether the various models predict vascular homeostasis, we analyzed the steady state response of the models to various changes in pressure and flow as well as to addition of vasoconstrictors and dilators. Three more models were tested, in which a single adaptation response was ‘knocked out’ (*FUNCTKO*: no tone regulation, *PLASTKO*: no SMC length adaptation, *REMODKO*: no change in slack radius). Four derived quantities with pathophysiological significance were included: wall stress and shear stress, radius reserve (ratio of fully dilated and actual inner radius) as an index of flow reserve, and tension match (the ratio of optimal inner radius for active tension development and passive inner radius at 100 mmHg). This ratio indicates how well active and passive biomechanics remain matched during remodeling. Figure 5 provides a color map of the sensitivity of these quantities to different input conditions. All four adaptation processes were required for maintenance of stable and functional vessels under different hemodynamic or biochemical conditions. Thus, in *GROWTH*, steady state effects of pressure on internal radius were absent, while flow, vasoconstrictors and vasodilators caused adaptations of internal radius that progressed from transient changes of tone to structural responses in steady state. These structural responses resulted in tone returning to control in steady state, ready to cope with additional stimuli. Additionally, SMC length was regulated, maintaining adequate force capacity when the radius changes chronically. Wall stress and shear stress were simultaneously regulated under changing pressure or flow. Wall stress was also regulated against addition of constrictors and dilators. However, shear stress in *GROWTH* increased respectively decreases in the presence of constrictors and dilators. Radius reserve and tension match were well regulated in *GROWTH*. Yet, *GROWTH* was not a fully stable model. For chronic high vasodilator concentrations, inner radius progressed towards infinity. At higher dilator concentrations, shear stress became lower. Their sum remained constant at 35 dynes/cm^2^, with instability occurring for dilator concentrations above this level.

Excluding one or more of the four adaptation processes resulted in severe impairment of adaptation. As examples, without SMC length regulation, not only SMC length but also tone and radius reserve became dependent on input conditions. Without eutrophic remodeling, tone and radius reserve were much more dependent on input levels, while radius was less sensitive to flow and vasoactive agents. *FUNCTKO* is not shown; all models with absence of tone regulation but intact structural regulation were found to be unstable, progressing towards either zero or infinite radius. This indicates that effects of mechanical loading on blood vessel tone are crucial for also long-term regulation, even though in *GROWTH* tone is unaltered in steady state.

### Parameter variation

**Figure 6:**
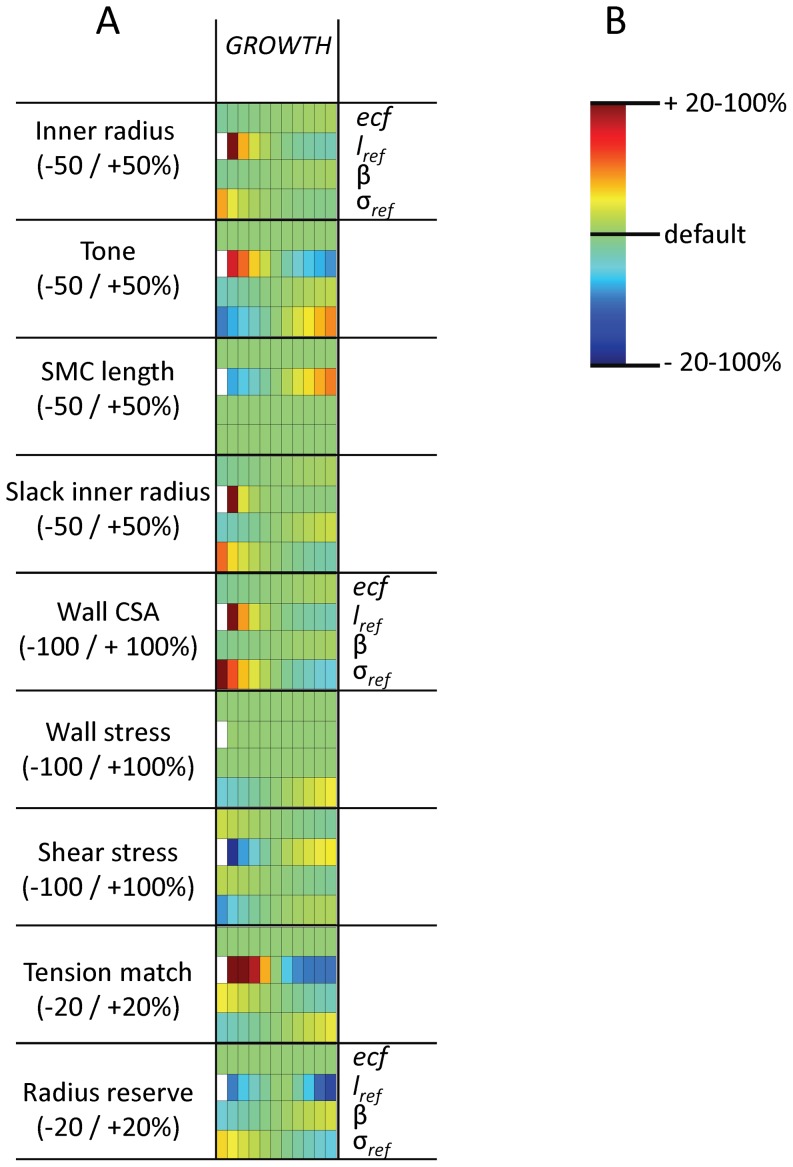
Regulation of the vessel state under parameter variation. A: Regulation of the vessel state and of additional quantities with pathophysiological significance under altered (–50% to +50%) model parameters. B: color scale for the model predictions. *ecf:* endothelial cell function, with *ecf* = 1 representing normal function and *ecf* = 0 full impairment of dilation. 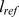 *:* reference length for SMC length adaptation. 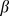: effect of strain on remodeling, with higher 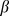 reflecting more outward remodeling.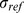: the reference stress for the growth response.

The model predictions are based on specific values for a range of parameters. Some of these could be estimated from experimental data, such as the parameterization of the matrix elasticity (Figure S1 in file S1). For others, notably in the adaptation processes, experimental data are not available or do not allow straightforward parameter estimation. We identified critical parameters in each of the adaptation processes and studied the effect of their variation on model outcome. Figure 6 shows these effects for *GROWTH*. Firstly, endothelial function is known to be crucial for tone regulation, and dysfunction is an important contributor to cardiovascular disease. In the model, better endothelial function resulted in a larger radius and decreased shear stress, but unchanged tone and radius reserve. Secondly, we addressed different reference lengths for SMC plasticity. This length was originally set to 80 microns, i.e. on the upslope of the length-tension relation. A larger reference length caused a much smaller radius, low tone and high shear stress. Radius reserve depended in a biphasic manner on reference length, with the default value of 80 micron resulting in near optimal reserve. The simultaneous high tone and low radius reserve that was found at low reference lengths reflects contractile dysfunction and forced dilation: the SMC are fully active but cannot generate sufficient tension to counteract the pressure. Thirdly, we considered an altered balance of tone and strain effects on matrix remodeling. Reducing the effect of strain on remodeling (β) caused a small decrease in radius, less tone and less vasodilator reserve, not unlike that seen in essential hypertensives (Figure S4B in file S1). Fourthly, raising the reference stress caused thinning of the wall (not shown), again leading to contractile dysfunction, with simultaneous maintenance of a normal radius. Under all parameter variations, shear stress remained independent of flow, indicating that such shear regulation occurred over a wide range of model parameters (Figure S6 in file S1). Table 3 provides the sensitivity of the model outcomes to parameter variation around their default values. As can be seen, all parameters but particularly the reference length for SMC length adaptation affected the internal radius, with large effects on tone, slack radius and wall CSA. EC dysfunction, the traditional factor considered in vascular pathology, had mild effects on diameter, with unchanged radius reserve.

**Table 3:** Sensitivity matrix for the effect of four model parameters on model outcome, represented by nine variables. Each cell indicates relative change in output divided by relative change in parameter.

	*ecf*	*l_ref_*	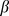	*σ_ref_*
*r_i_*	0.150	–0.567	0.124	–0.222
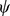	0.000	–1.374	0.296	0.952
*l*	0.000	1.000	0.000	0.000
*r_islack_*	0.150	–0.144	0.340	–0.410
*wCSA*	0.301	–1.131	0.249	–1.576
*σ*	0.000	0.000	0.000	1.000
*τ*	–0.450	1.720	–0.372	0.668
*tension match*	0.000	–1.405	–0.165	0.176
*radius reserve*	0.000	0.225	0.168	–0.237

## Discussion

The current study shows how several processes together maintain resistance vessels in an optimal state during their adaptation towards a new caliber in response to a range of stimuli. These are wall mechanics and four biological adaptation processes: regulation of tone, rearrangement of SMC and of matrix, and a trophic response. This full model, GROWTH, was stable for all stimuli with the exception of continuous strong vasodilation. The maintained optimal state was characterized by regulated wall stress and shear stress, intermediate tone and normal vascular reserve, and matching of active and passive biomechanics. In this state, the vessel is ready to cope with new challenges, allowing continuous long-term vasoregulation. Excluding any of the four biological processes resulted in a poorly regulated state and in some cases instability of vascular structure.

### Included processes

Most relations in the model were based on extensive experimental work on small arteries, using a variety of in vitro and in vivo interventions and genetic models (see data in file S1).

**Mechanics.** We used a bi-exponential stress-strain relation for the vascular matrix without making assumptions on the contribution of elastin and collagen. In more elaborate adaptation models that include differential growth or reorganization of elastin and collagen, such a separation will be needed.

**Tone control.** Central in the model was regulation of tone. The sensitivity to four stimuli was based on experimental data on pressure and flow effects [28], the observation that pressure and vasoconstrictors act synergistically over some range [29], and the role of nitric oxide and other EC-derived factors as common pathways in shear and vasodilator effects [15]. For these reasons the vasoactive agent effects were expressed as equipotent wall stress and shear stress. Inclusion of specific vasoactive agents and their concentrations would have been relatively straightforward, but would provide little additional insight.

The central role of tone has previously been considered in a model study by Jacobsen *et al.* [13]. These authors considered the case for eutrophic remodeling, excluding growth. In their model, a change in pressure initially results in a myogenic response. This change in tone then drives remodeling, such that the vascular wall returns to a homeostatic state of stress, strain, and activation after a sustained change in pressure. The authors did not include flow. Our predictions in *REMOD* (i.e. without growth) show that also under flow, elevation of pressure would cause structural narrowing if hypertrophy were absent. Yet, in *GROWTH* such inward remodeling did not occur.

**SMC length regulation.** Evidence for SMC rearrangement comes from studies on SMC relengthening [18] as well as from altered active radius-tension relationships after in vivo flow interventions [30] and following chronic vasoconstriction in *in vitro* vessels [19]. While we included SMC adaptation based on regulation of cell length, an alternative mechanism could be adaptation of the cell cytoskeleton to cell length, as was suggested for airway SMC [31]. This could however be implemented by comparable relations.

**Remodeling and growth.** Changes in vascular structure have two aspects: remodeling and growth. Depending on the stimuli, either aspect may dominate the final structural and biomechanical outcome. In the model, we separated remodeling and growth because of the different stimuli. Remodeling of both SMC and matrix was taken as eutrophic by definition. Yet, in the absence of growth there still may be a balance of deposition and breakdown of matrix, where the embedding of new matrix depends on strain and tone. It is appreciated that matrix synthesis and degradation are separate processes with their own signaling and enzymes, and that this also holds for cellular hypertrophy and hypotrophy respectively proliferation and apoptosis. Our model only considered the balances without making assumption on which arm of this balance is influenced by the various stimuli. We did not specify whether SMC growth was hypertrophy or hyperplasia. We lumped growth of matrix and cells into a single process, in which the relative composition of the wall was maintained and in which the stress-strain relations were unaffected. We thus ignored possible differential growth and altered biomechanical properties during growth, related to phenotypic changes of SMC and maturation of the matrix. The initial composition of passive, active and cytoskeletal elements in equal thirds was taken arbitrarily but other values would provide identical results if at the same time the stress-strain relations are rescaled.

Remodeling was driven by a misbalance of strain and tone (eq. 18). We varied only the sensitivity to strain (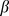) in this relation; since only the balance is relevant, opposite variation of the sensitivity to tone would have given comparable results. In fact, eq. 18 and several other equations have more parameters than strictly needed. However, this way the parameters are more meaningful than in the case of a minimized, often dimensionless set of parameters.

We limited the role of chemical stimuli to their effect on tone, and ignored a possible direct influence of e.g. cytokines on any of the three structural adaptation processes. For remodeling and SMC plasticity, we feel that this is a defendable choice: while many processes have been identified that influence matrix remodeling and potentially SMC plasticity, the inward versus outward direction seems to depend primarily on the local mechanics resulting from external load and traction forces associated with tone. We experimentally demonstrated that large changes in flow in the rat mesenteric bed cause upregulation of many inflammatory pathways. However, such upregulation was similar at increased and decreased flow, while tone and the direction of remodeling were clearly different [11]. From that work we concluded that inflammation is an essential part of the remodeling process, but that tone drives the inward or outward direction of such remodeling. Inflammation could have been included in the model as a higher remodeling speed, but this would not have affected the steady state predictions. Activation of specific enzymes does potentially lead to inward or outward remodeling independent of tone, even though classification of enzymes as ‘inward’ (transglutaminases [25]) or ‘outward’ (matrix metalloproteinases) is oversimplifying [32]. Such actions, as well as the trophic response to e.g. angiotensin II [33], could have been incorporated as a changed balance of tone and distension or altered reference stress for growth.

### Single segment versus network approaches

We simulated a single segment, excluding feedback from the perfused tissue and network properties. Levels of pressure, flow, and vasoactive agents were therefore constant inputs. This is clearly artificial, but provided insight into the relation between functional and structural responses of the vessel. It should be stressed that the maintenance of an optimal state during adaptation reflects the vessel wall itself, rather than optimal function in terms of flow and oxygen delivery. In e.g. the presence of vasoconstrictors in GROWTH, all aspects of the vessel scaled down to a smaller size, leaving a vessel that may provide insufficient flow to the perfused tissue. Feedback by tissue-derived signals is needed to drive this vessel scale. This could be implemented by making *CON* or *DIL* dependent on the balance of oxygen supply and demand. In a next step, network approaches should be applied, where pressure and flow are no longer independent constants. More complex networks would include multiple instances of the current model segment, arranged in branching or arcading patterns, with possible addition of direct communication as an important signal.

A specific aspect of the ‘single segment’ choice relates to the regulation of shear stress. We found that flow caused an increase in diameter such that shear stress remained unchanged. Yet, in a network the increased vessel conductance would again cause a higher flow and shear stress. This could result in a fundamentally different response of the network versus the single segment, including possible instability. In a previous theoretical study, we demonstrated that at least part of the elements should have shear-independent resistance in order to accomplish network stability [34]. Alternatively, feedback from the perfused tissue, via vasoactive agents (*CON* and *DIL* in the model) may be critical in maintaining stability in networks. Such feedback would still change the level of shear stress: in our model predictions, shear stress became higher in the presence of vasoconstrictors and lower with vasodilators. Such influences could also explain the differences in shear stress seen in major arteries versus arterioles and veins [35]. These issues need to be addressed in future work.

### Model behavior

GROWTH demonstrates interactions that are not always appreciated in experimental work. These include the interdependent regulation of tone and structure, as well as the interplay between pressure and flow effects on tone and structure. Thus, high flow caused wall hypertrophy due to the raised wall stress upon vasodilation (Laplace relation) rather than by direct shear-induced trophic responses. We realize that there are many remarks to make on the modeling choices. Yet, such interactions do occur, with consequences for the interpretation of experimental data at e.g. the level of gene expression and cell signaling. We would therefore argue that a more integrated approach is needed in also experimental work on resistance arteries.

GROWTH became unstable for high vasodilator concentrations, progressing to an aneurysm-like state. Critical here is that the progression of slack radius depended on the balance of distension and tone (eq. 18). The effect of distension on slack radius was based on continuous matrix or cross-bridge turnover, where new material is deposited at the actual distension. The role of tone here was based on the observation that active tone rather than only a lack of distension induces inward remodeling in small arteries [23]. Vasodilation strongly shifts this balance by reducing tone and increasing distension. Alternatives for eq. 18 could have been considered, and it remains to be seen if these lead to better stability against vasodilator actions. Yet, experimental evidence for rapid and substantial vasodilator-induced outward remodeling comes from cultured coronary vessels in the presence of an L-type calcium channel blocker [24]. In addition, mutations in SMC alpha-actin, myosin heavy chain and myosin light chain kinase have been associated with dissections and aneurysms [36]–[38], indicating that contractile activity is indeed needed to balance outgrowth and maintain vascular structure.

### Relevance for cardiovascular disease

Essential hypertension is characterized by a relatively unchanged cardiac output and consequently a matched rise in peripheral resistance and systemic blood pressure. Moreover, flow reserve is very strongly impaired in hypertension, pointing at a large structural reduction in resistance vessel radius [4], compensated by a state of relative vasodilation [21]. The etiology is still poorly understood. Several stimuli and model alterations caused inward remodeling in GROWTH, including extrinsic vasoconstrictors and endothelial dysfunction. These are commonly considered as causes of increased peripheral resistance. Yet, in both cases, relative vascular radius reserve (ratio of dilated and active radius) remained intact. Two tested perturbations caused simultaneous reduction in radius, radius reserve and tone, possibly reflecting the small artery state in hypertension: The first one was an increase in reference for SMC length adaptation, or more generally a relative rightward shift of this reference length with respect to the active length-tension relation. This balance has not been experimentally studied in blood vessels. In airway SMC, alterations in this balance have been implied in asthma [31] and in analogy a similar role for vascular SMC could exist in hypertension. The second perturbation was a reduction of the effect of strain on outward remodeling, or more generally a smaller ratio of strain-induced outward and tone-induced inward remodeling tendencies (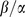 in equation 18). Experimental tests of these predictions are feasible and may improve the understanding of part of the complex etiology of hypertension.

### Conclusion

In conclusion, this quantitative and integrative approach provides insight into the functional and structural regulation of resistance vessel caliber. This study indicates how vessels maintain their stability and functionality while adapting to pressure, flow and vasoactive agents. It helps putting experimental data into perspective, and helps unraveling causes and consequences of cardiovascular pathologies. Finally, the current model could form a module in more extensive computational approaches in medicine, including Human Physiome initiatives [39].

## Supporting Information

File S1 **Experimental evidence and analyses that form the base for the modeling choices in the current study (figures S1 to S5).** Three-dimensional representation of the effects of parameter variation at various flows (figure S6).
(PDF)
