# Supplementary material for: Integrative Modeling of Small Artery Structure and Function Uncovers Critical Parameters for Diameter Regulation
Source: PLoS One. 2014 Jan 31;9(1):e86901. doi: 10.1371/journal.pone.0086901 (PMC3908953; doi:10.1371/journal.pone.0086901)
Supplement: File S1 — Experimental evidence and analyses that form the base for the modeling choices in the current study (figures S1 to S5). Three-dimensional representation of the effects of parameter variation at various flows (figure S6). (PDF) [file pone.0086901.s001.pdf]

Supporting information

Below we provide experimental evidence and analyses that form the base for the modeling choices in the current study.

Mechanics: Vascular wall composition and mechanical loading

Figure S1A-C depicts the predicted passive, maximal active and cytoskeletal stresses as a function of midwall radius for the default settings in steady state. Figure S1D provides inner radius-tension relations for a range of steady tones (dashed blue), reflecting the pure mechanical responses to distension. Such relations can experimentally be verified at full vasodilation (tone=0, solid blue) and full activation (tone=1, solid brown) on respectively wire-mounted (S1E) and cannulated (S1F) small arteries. Two experimental datasets are indicated here. The red data points in S1D were obtained on wire-mounted mesenteric vessels [1]. The green circles in S1D were obtained on cannulated rat mesenteric vessels, partly subjected to isometric loading by feedback adaptation of the pressure [2], and converted based on the Laplace relation. These experimental data show similar qualitative behavior, although there are quantitative differences. For the passive biomechanics, the model was tuned to predict a steady state under default conditions that fits the passive characteristics determined on the cannulas, as this is a very robust measurement. For the biomechanics at full activation (brown solid), an intermediate relation was sought between the two datasets. Figure S1G shows the predicted pressure-radius relation, linked to S1D by the Laplace relation. These data and model predictions show the classic biomechanics: a passive relation with increased stiffness at higher distension and decreased compliance at higher pressure, and relations at full activation characterized by increased tension-generating capacity at distensions up to an optimal radius that is close to the maximal passive radius. A consequence of this shape is that radius of blood vessels operating at intermediate tone is extremely sensitive to pressure variation, as was shown in figure 3 in the main manuscript, requiring rapid adaptation of tone to pressure.

Adaptation 1: smooth muscle cell contractile activity (tone)

Figure S2A demonstrates the adaptation of tone to pressure, the myogenic response. Shown are experimentally derived data on luminal cross-sectional area, obtained on cannulated rat mesenteric vessels [3]. In particular *in* vitro mesenteric vessels, basal tone is not always present, requiring (unlike *in vivo*) extra stimulation, in this case by 30 nM of the L-type calcium channel opener BAYK8644. In its presence, vessels respond to a pressure increase by a rapid distension followed by a rise in tone and consequent vasoconstriction (green), as opposed to the pure mechanical distension in the passive

state (blue). We used an interpolation between two other datasets on mesenteric artery myogenic responsiveness[2] to tune the model (Figure S1H: blue squares: tone under basal conditions, green squares: tone in the presence of 1 µM phenylephrine, pink triangles: model). Note that the myogenic response renders radius essentially independent of pressure over a large working range (S1G pink (*FUNCT* model) versus dotted blue (*MECH* model) relations). A slight negative slope of the pressure-diameter is present. This slope may suggest a rather weak myogenic response. However, unlike the isolated vessel example in figure S2A, the simulation was made in the presence of constant flow, where shear effects are opposing the myogenic response. The strength of the myogenic response or the presence or not of a negative myogenic pressure-diameter relation did not fundamentally affect the model predictions in the current study (data not shown).

Figure S2B shows an experimental study on the functional effect of flow, in this case on cannulated porcine coronary vessels [4]. An increase in pressure gradient between left and right cannula induced flow and shear stress without a change in luminal pressure. This induced a vasodilation, and consequently a reduction of shear stress. Based on such data, a shear-dependent dilation was incorporated into the model.

Adaptation 2: SMC span and length (SMC plasticity)

Figure S3A collects data on SMC length as function of vascular diameter. These data indicate that SMC length is essentially identical in large and small vessels, pointing towards mechanisms for regulation of cell length during vascular adaption and growth. Data from Martinez-Lemus *et al.* indicate that such reorganization and relengthening is very fast [5]. Thus, noradrenaline induced vessel constriction and SMC shortening, which were reversible after 30 minutes (open symbols in S3B). Yet, under maintained stimulation, SMC relengthened in the course of 240 minutes (red symbols). Upon subsequent washout, cell length returned to control but the vessel diameter remained smaller (closed symbols). This uncovers a cytoskeletal component to passive stress, preventing full recovery of diameter after vasodilation. This prompted us to include a cytoskeletal element in the vessel wall (see Figure S1C). Indeed, our model predicts impaired vasodilation in the transient phase following vasoconstriction and plasticity but before onset of matrix remodeling (not shown). A further consequence of SMC plasticity is the altered active diameter-tension relation, since for a given diameter the SMC have a different length. Figure S3C provides experimental evidence on rat mesenteric vessels, mounted on isometric wires. These vessels were maintained overnight at a low distension in the presence of endothelin-1, resulting in a strong inward shift of the force generating capacity, such that the tension match (the ratio of optimal diameter for tension development and passive diameter at 100 mmHg) was significantly reduced, and vessels could

generate much more tension at small diameters [1]. Similar results were obtained in *in vivo* experiments [6].

Adaptation 3: eutrophic matrix remodeling

Eutrophic matrix remodeling reflects reorganization of the matrix around a smaller or larger lumen, with unchanged wall CSA but changed wall thickness (Figure S4A). Such remodeling may occur in response to a range of stimuli, and is a hallmark of hypertension. The large consequences in hypertension are demonstrated in Figure S4B. In established essential hypertension, cardiac output is virtually unchanged. Consequently, the peripheral resistance increases in proportion to the blood pressure, shown for grade I and grade II hypertension in the coronary and forearm circulation (dark blue, dark green). Yet, the minimum resistance of both vascular beds, obtained during full vasodilation, has increased much stronger (light blue light green), up to more than 100% in the coronary circulation. This is believed to result from a strong inward remodeling, partly compensated by continuous vasodilation in order to maintain adequate flow during autoregulation.

Various experimental models for small artery matrix remodeling exist. In figure S4C, low flow and high flow branches in the arcading mesenteric circulation were created by local obstructions (left). These vessels have initial tone before the occlusion (S4D, C_N versus D_N). The occlusion then causes a deeper constriction in the low-flow branches, as determined after 2 hours (C_{LF}). In contrast, the high flow branches obtained a more vasodilated state (C_{HF}). After two days, this functional response was transmitted towards structural remodeling, demonstrated by the lower and higher passive diameter in low flow and high flow vessels, respectively (open bars). In several studies, we found that active tone is an important drive for inward matrix remodeling. Figure S4E shows an example for cannulated cremaster muscle arterioles kept in organoid culture at 75 mmHg [7]. These vessels developed deep tone in the presence of intravascular fetal calf serum, which resulted in a reduction of the passive diameter (tested at 100 mmHg) after 3 days (group 1). Tone and such inward remodeling were fully prevented by a calcium blocker (group 2). Vessels maintained at only 5 mmHg during culture maintained a small diameter due to collapse, but had no tone due to the myogenic response. Also those vessels did not remodel, despite the presence of growth factors (group 3). A substantial set of studies has since then shown such coupling between tone and matrix remodeling, prompting us to model eutrophic remodeling as a misbalance between a tone-based inward drive and a strain-based outward drive.

Adaptation 4: wall cross-sectional area growth

Larger vessels have thicker walls, and it is generally believed that wall stress is an important drive in the growth of wall CSA. Thus, the initially eutrophic outward remodeling as described in Figure S4C

for two days of high flow, has been found to be followed by a hypertrophic response in the subsequent four weeks (figure S5A). For low flow, the reverse process occurs. Figure S5B illustrates that such trophic responses help normalizing the wall stress. In this specific dataset, comparing the data with the iso-stress curve (dashed line, Laplace assuming constant pressure) reveals that the low flow response maintains wall stress fully normalized by reduction of both internal diameter and wCSA, while the trophic response at high flow at this time point has not yet normalized wall stress [8]. Observations like these were accounted for by a final slow adaptation of wall CSA such that wall stress is normalized.

In hypertension, based on the Laplace relation, vessels may normalize wall stress by either inward remodeling or hypertrophy. Small arteries differ from main vessels in this respect. Thus, large vessels demonstrate primarily wall hypertrophy [9]. This is illustrated in S5C-D, where during development inner diameter of the aorta grows similarly in spontaneously hypertensive rats (SHR) and control Wistar-Kyoto rats (WKY), while wCSA increases much more in the SHR animals. In contrast, small arteries in hypertension mainly exhibit eutrophic inward remodeling, such as reflected in Figure S4. The causes of this disparity are unknown, but may relate to inward remodeling being a cause of hypertension more than a consequence of wall stress adaptation. In the model, both remodeling and growth were incorporated, their relative response to pressure changes being an emerging property of the model.

Legends supplemental figures

Figure S1: Model analysis and experimental data on basic biomechanical relations. The model data were obtained by perturbations from *GROWTH* steady state under default conditions. A-C: midwall radius-stress relations of the three stress-bearing elements predicted by *MECH*. D: internal radius-tension relation as would have been measured on an isometric wire myograph. Solid blue: *MECH*, ψ =0. Dotted blue: *MECH*, ψ = 0.2, 0.4, 0.6, 0.8. Solid brown: ψ=1. Green: data from cannulated vessels [2]. Red: data from wire-mounted vessels [1]. Black dash-dotted line: Laplace relation at 100 mmHg. E: image and schematic drawing of the wire myograph. F: microscopic image of a cannulated vessel, pressurized from both ends. G: *MECH*, pressure-radius relations analogue to S1D; pink: *FUNCT*, myogenic adaptation to pressure. H: pink: *FUNCT* myogenic adaptation to pressure, in the presence (green) and absence (blue) of 1 µM phenylephrine (data from [2]).

Figure S2: Experimental evidence for functional adaptation. Shown are responses to pressure (A; blue: passive, green: myogenic response) and flow (B), as determined on cannulated small arteries (figure S1F). Figures based on data from [3] (A) and [4] (B).

Figure S3: Experimental evidence for SMC plasticity. A: SMC length versus vascular diameter. Source of data is indicated in the table below. B: Normalized SMC length versus diameter during 5 and 240 minutes stimulation with noradrenaline, based on data from [5]. C: Active tension capacity shifts to smaller diameters after 1 day stimulation with 100 nM endothelin-1 while maintaining the vessel at a normalized diameter of 0.4. Based on data from rat mesenteric small arteries, with diameter normalized to the passive value at 100 mmHg [1].

symbol in S3A	vessel source	intervention/group	reference
black filled circle	WKY rat intestinal	young WKY	[10]
black open circle	WKY rat intestinal	old WKY	[10]
red open square	WKY rat mesenteric	low flow WKY	[11]
red/orange square	WKY rat mesenteric	normal flow WKY	[11]
red filled square	WKY rat mesenteric	high flow WKY	[11]
green triangle	WKY rat mesenteric	control	[12]
purple circle	WKY rat mesenteric	control	[13]
red diamond	WKY rat mesenteric	control	[14]
blue filled diamond	hamster cheek pouch	control	[15]
brown circle	hamster cheek pouch	control	[16]
open blue diamond	Sprague–Dawley rat pia	control	[15]
black square	WKY rat aorta	isolated cells	[17]

Figure S4: Experimental evidence for eutrophic remodeling. A: schematic drawing of eutrophic inward and outward remodeling. B: Minimal resistance (Rmin) increases disproportionally in hypertension, causing impairment of flow reserve. EH: essential hypertension, MAP: mean arterial pressure. Data from [18]. C: creation of high (100% flow increase) and low flow vessels. D: Passive and active diameters following flow intervention (D_N: dilated control vessels, C_N: constricted, basal tone and normal flow, C_{LF} and C_{HF}: constriction levels after 2 hours of low and high flow, D_{LF} and D_{HF}: dilated diameter after 2 days of low flow and high flow). *: P<0.05, effects of flow on active diameter; #: P<0.05, D_{LF} versus D_{HF}. Data from [19]. E: Long-lasting tone induces inward remodeling, which is prevented by verapamil or by incubation at low pressure. Shown are diameters after incubation, at full relaxation and 100 mmHg. Data from [7].

Figure S5: Experimental evidence for trophic responses. A: schematic drawing of hypertrophy following eutrophic outward remodeling. B: changes in passive diameter and wall CSA after 4 weeks of flow intervention, demonstrating remodeling. Open square: sham controls, black square: low flow, red square: high flow. Dashed line: constant wall stress. Data from [8]. C, D: Carotid artery

remodelling during development (black squares: young, red squares: old) in WKY (open symbols) and SHR rats(closed symbols). Data from[9]. *: $P<0.05$, WKY versus SHR.

Figure S6: Effect of parameter variation. Shown are inner radius, tone, shear stress and radius reserve as function of flow and of four parameters: A: Endothelial function, with *ecf*=1 representing normal function and *ecf*=0 full impairment of dilation; B: reference length (l_{ref}) for SMC length adaptation; C: effect of strain on remodeling, with higher β reflecting more outward remodeling; D: the reference stress (σ_{ref}) for the growth response.

Reference List

1. Tuna BG, Schoorl MJ, Bakker EN, De Vos J, vanBavel E (2013) Smooth muscle contractile plasticity in rat mesenteric small arteries: sensitivity to specific vasoconstrictors, distension and inflammatory cytokines. Journal of Vascular Research in press.
2. VanBavel E, Wesselman JP, Spaan JA (1998) Myogenic activation and calcium sensitivity of cannulated rat mesenteric small arteries. Circ Res 82: 210-220.
3. Wesselman JP, VanBavel E, Pfaffendorf M, Spaan JA (1996) Voltage-operated calcium channels are essential for the myogenic responsiveness of cannulated rat mesenteric small arteries. J Vasc Res 33: 32-41.
4. Sorop O, Spaan JA, Sweeney TE, VanBavel E (2003) Effect of steady versus oscillating flow on porcine coronary arterioles: involvement of NO and superoxide anion. Circ Res 92: 1344-1351.
5. Martinez-Lemus LA, Hill MA, Bolz SS, Pohl U, Meininger GA (2004) Acute mechanoadaptation of vascular smooth muscle cells in response to continuous arteriolar vasoconstriction: implications for functional remodeling. FASEB J 18: 708-710.
6. Tuna BG, Bakker EN, Vanbavel E (2013) Relation between active and passive biomechanics of small mesenteric arteries during remodeling. J Biomech 46: 1420-1426.
7. Bakker EN, van der Meulen ET, van den Berg BM, Everts V, Spaan JA, et al. (2002) Inward remodeling follows chronic vasoconstriction in isolated resistance arteries. J Vasc Res 39: 12-20.
8. Pourageaud F, De Mey JG (1997) Structural properties of rat mesenteric small arteries after 4-wk exposure to elevated or reduced blood flow. Am J Physiol 273: H1699-1706.
9. Cebova M, Kristek F, Kunes J (2006) Differential remodeling of carotid artery in spontaneously hypertensive and hereditary hypertriglyceridemic rats. Physiol Res 55 Suppl 1: S81-87.
10. Miller BG, Connors BA, Bohlen HG, Evan AP (1987) Cell and wall morphology of intestinal arterioles from 4- to 6- and 17- to 19-week-old Wistar-Kyoto and spontaneously hypertensive rats. Hypertension 9: 59-68.
11. Buus CL, Pourageaud F, Fazzi GE, Janssen G, Mulvany MJ, et al. (2001) Smooth muscle cell changes during flow-related remodeling of rat mesenteric resistance arteries. CircRes 89: 180-186.
12. Korsgaard N, Mulvany MJ (1988) Cellular hypertrophy in mesenteric resistance vessels from renal hypertensive rats. Hypertension 12: 162-167.
13. Mulvany MJ, Baandrup U, Gundersen HJ (1985) Evidence for hyperplasia in mesenteric resistance vessels of spontaneously hypertensive rats using a three-dimensional disector. Circ Res 57: 794-800.

14. Rizzoni D, Porteri E, Piccoli A, Castellano M, Bettoni G, et al. (1998) Effects of losartan and enalapril on small artery structure in hypertensive rats. Hypertension 32: 305-310.
15. Haas TL, Duling BR (1997) Morphology favors an endothelial cell pathway for longitudinal conduction within arterioles. Microvasc Res 53: 113-120.
16. Walmsley JG, Gore RW, Dacey RG, Jr., Damon DN, Duling BR (1982) Quantitative morphology of arterioles from the hamster cheek pouch related to mechanical analysis. Microvasc Res 24: 249-271.
17. Murphy JG, Khalil RA (2000) Gender-specific reduction in contractility and [Ca(2+)](i) in vascular smooth muscle cells of female rat. Am J Physiol Cell Physiol 278: C834-844.
18. Eftekhari A, Mathiassen ON, Buus NH, Gotzsche O, Mulvany MJ, et al. (2011) Disproportionally impaired microvascular structure in essential hypertension. J Hypertens 29: 896-905.
19. Bakker EN, Pistea A, Spaan JA, Rolf T, de Vries CJ, et al. (2006) Flow-dependent remodeling of small arteries in mice deficient for tissue-type transglutaminase: possible compensation by macrophage-derived factor XIII. Circ Res 99: 86-92.

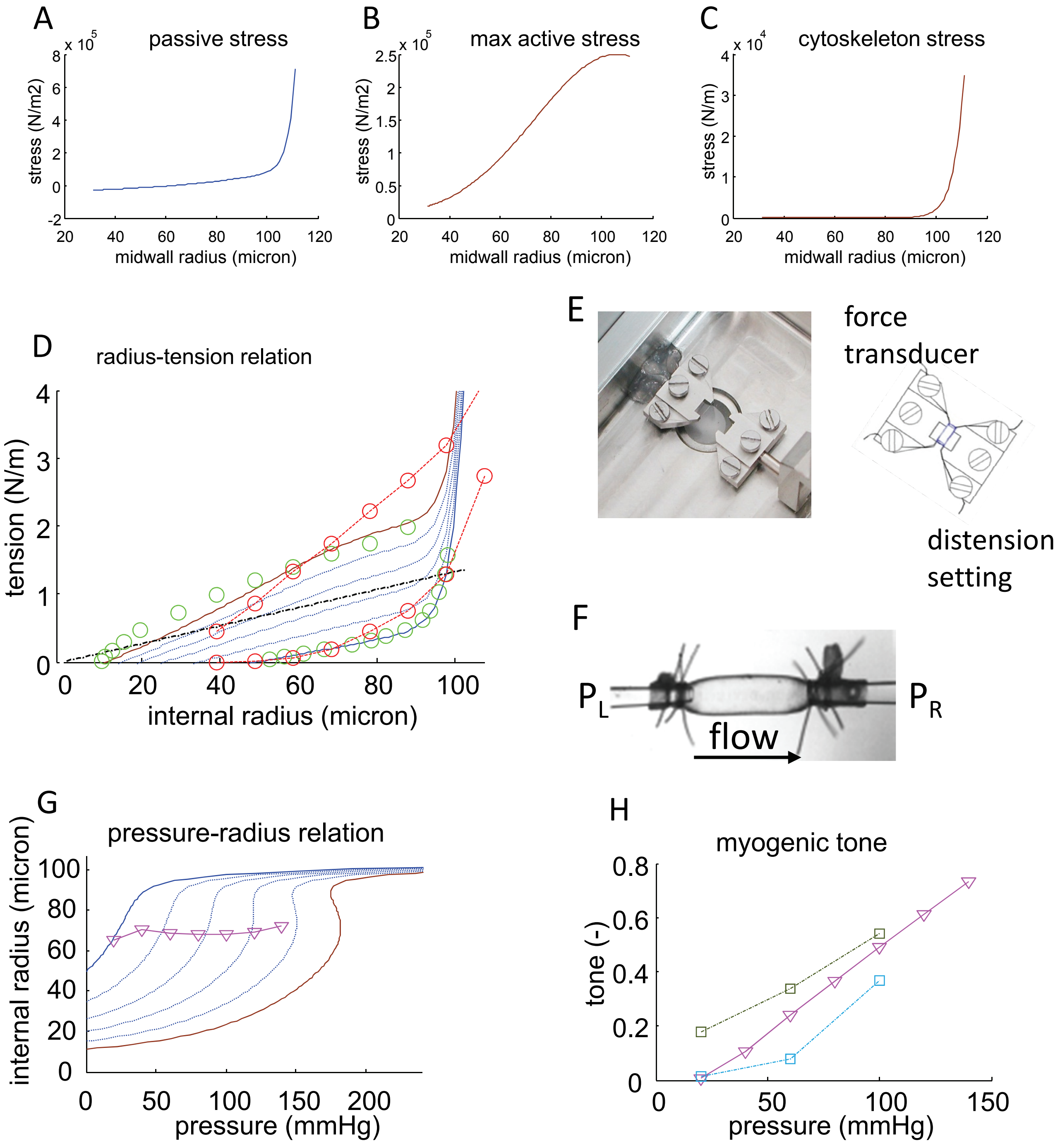
A
passive stress
x 10^5
stress (N/m2)
midwall radius (micron)
B
max active stress
x 10^5
stress (N/m2)
midwall radius (micron)
C
cytoskeleton stress
x 10^4
stress (N/m)
midwall radius (micron)
D
radius-tension relation
tension (N/m)
internal radius (micron)
E
force transducer
distension setting
F
P_L
P_R
flow
G
pressure-radius relation
internal radius (micron)
pressure (mmHg)
H
myogenic tone
tone (-)
pressure (mmHg)

Fig S1

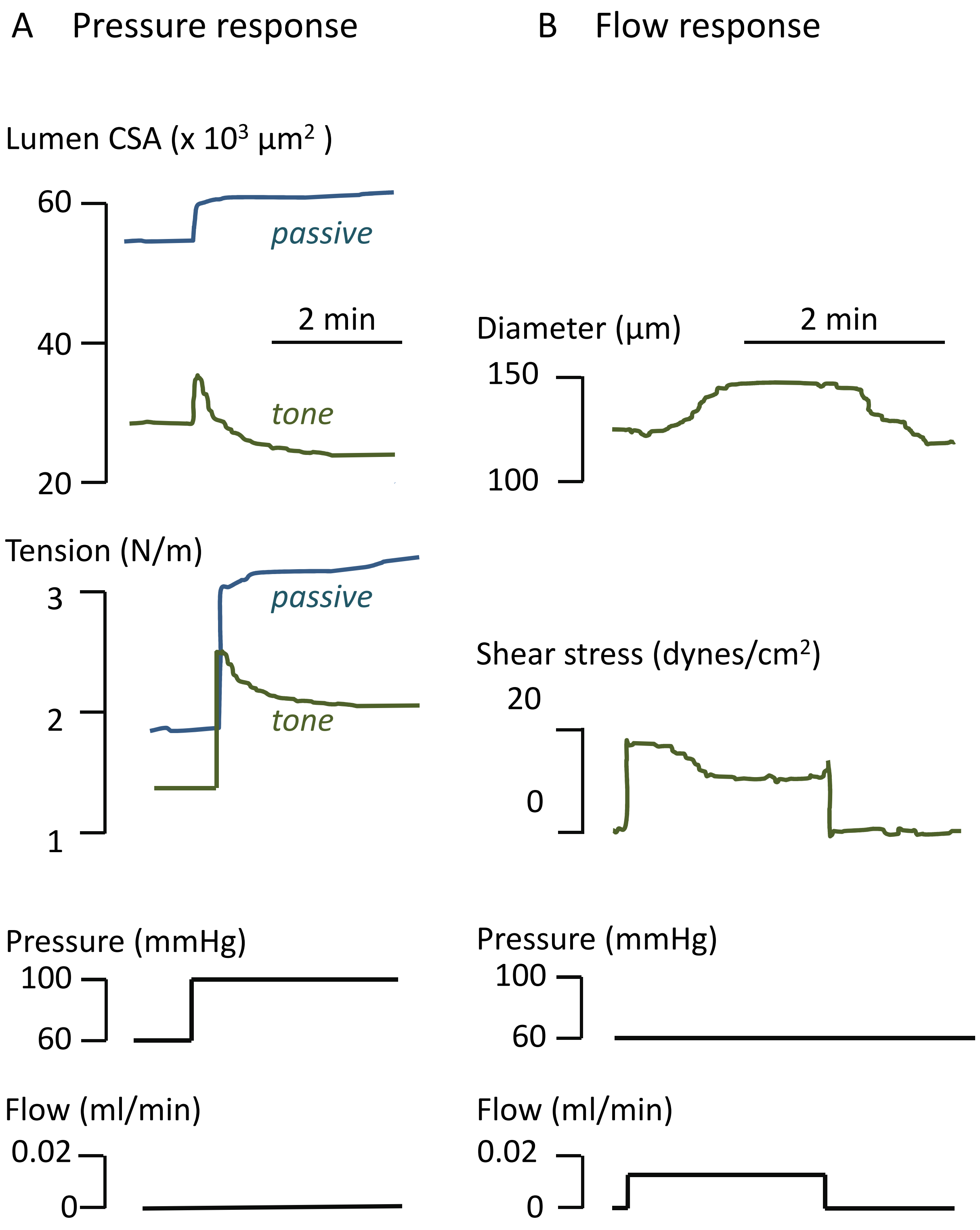
A Pressure response
B Flow response
Lumen CSA (x 10³ µm²)
60
40
20
passive
2 min
tone
Tension (N/m)
3
2
1
passive
tone
Pressure (mmHg)
100
60
Flow (ml/min)
0.02
0
Diameter (µm)
2 min
150
100
Shear stress (dynes/cm²)
20
0
Pressure (mmHg)
100
60
Flow (ml/min)
0.02
0

Fig S2

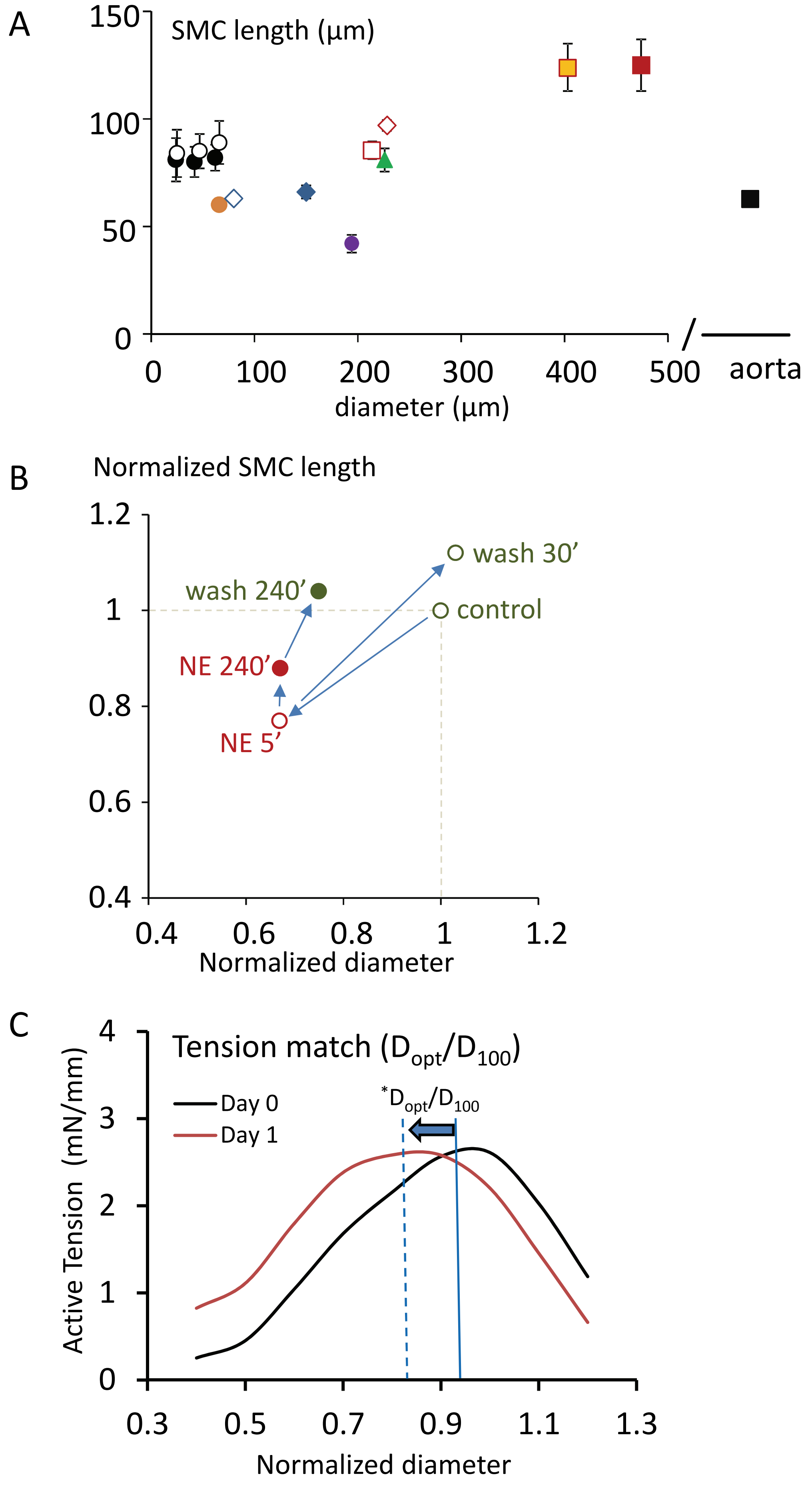
A
SMC length (µm)
150
100
50
0
0
100
200
300
400
500
aorta
diameter (µm)
B
Normalized SMC length
1.2
1
0.8
0.6
0.4
0.4
0.6
0.8
1
1.2
Normalized diameter
wash 240'
wash 30'
control
NE 240'
NE 5'
C
Active Tension (mN/mm)
4
3
2
1
0
Tension match (Dopt/D100)
Day 0
Day 1
*Dopt/D100
0.3
0.5
0.7
0.9
1.1
1.3
Normalized diameter

Fig S3

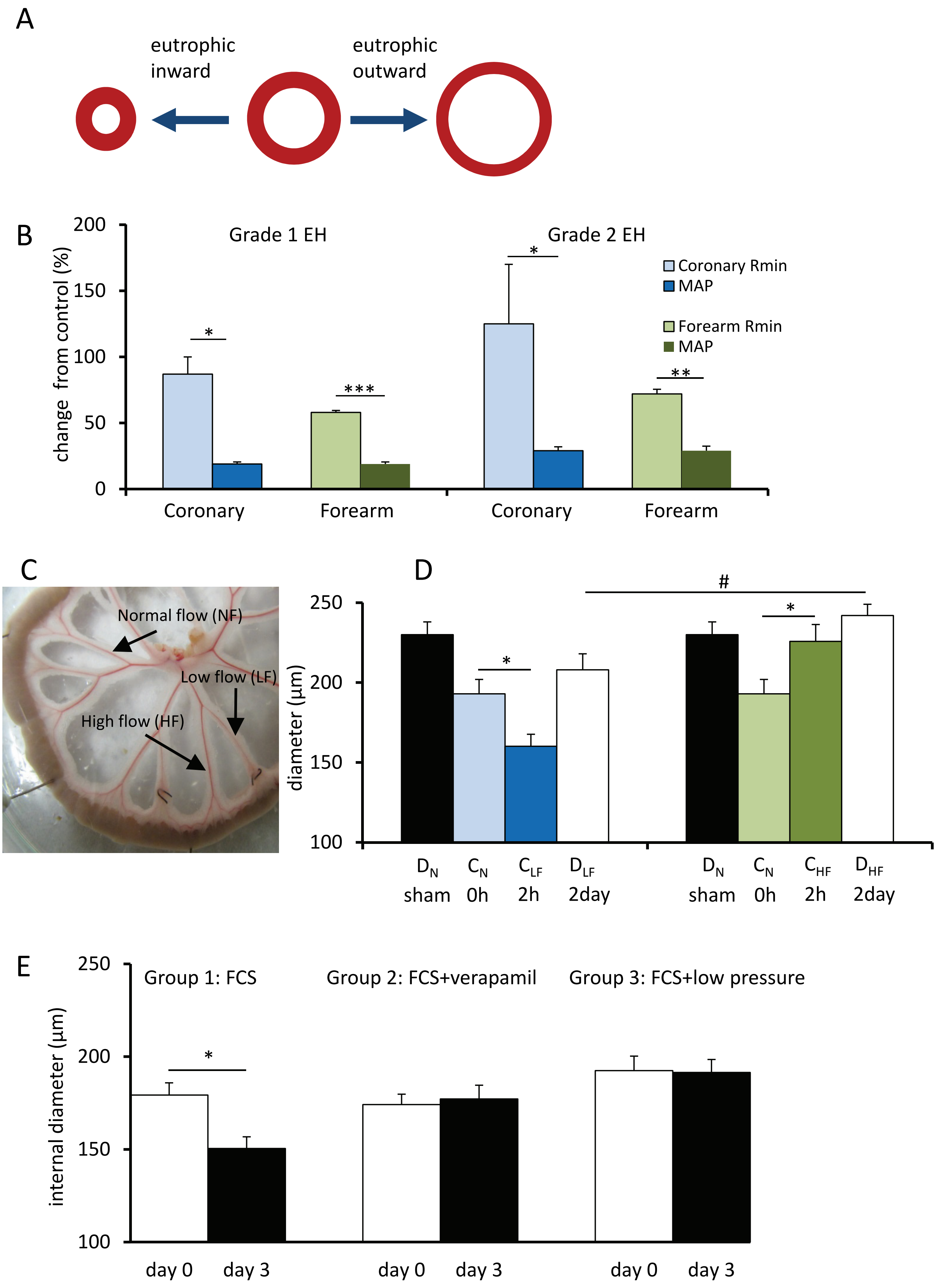
A
eutrophic inward
eutrophic outward
B
Grade 1 EH
Grade 2 EH
change from control (%)
200
150
100
50
0
*
***
*
**
Coronary Rmin
MAP
Forearm Rmin
MAP
Coronary
Forearm
Coronary
Forearm
C
Normal flow (NF)
Low flow (LF)
High flow (HF)
D
#
*
*
diameter (µm)
250
200
150
100
D_N sham
C_N 0h
C_{LF} 2h
D_{LF} 2day
D_N sham
C_N 0h
C_{HF} 2h
D_{HF} 2day
E
Group 1: FCS
Group 2: FCS+verapamil
Group 3: FCS+low pressure
internal diameter (µm)
250
200
150
100
*
day 0
day 3
day 0
day 3
day 0
day 3

Fig S4

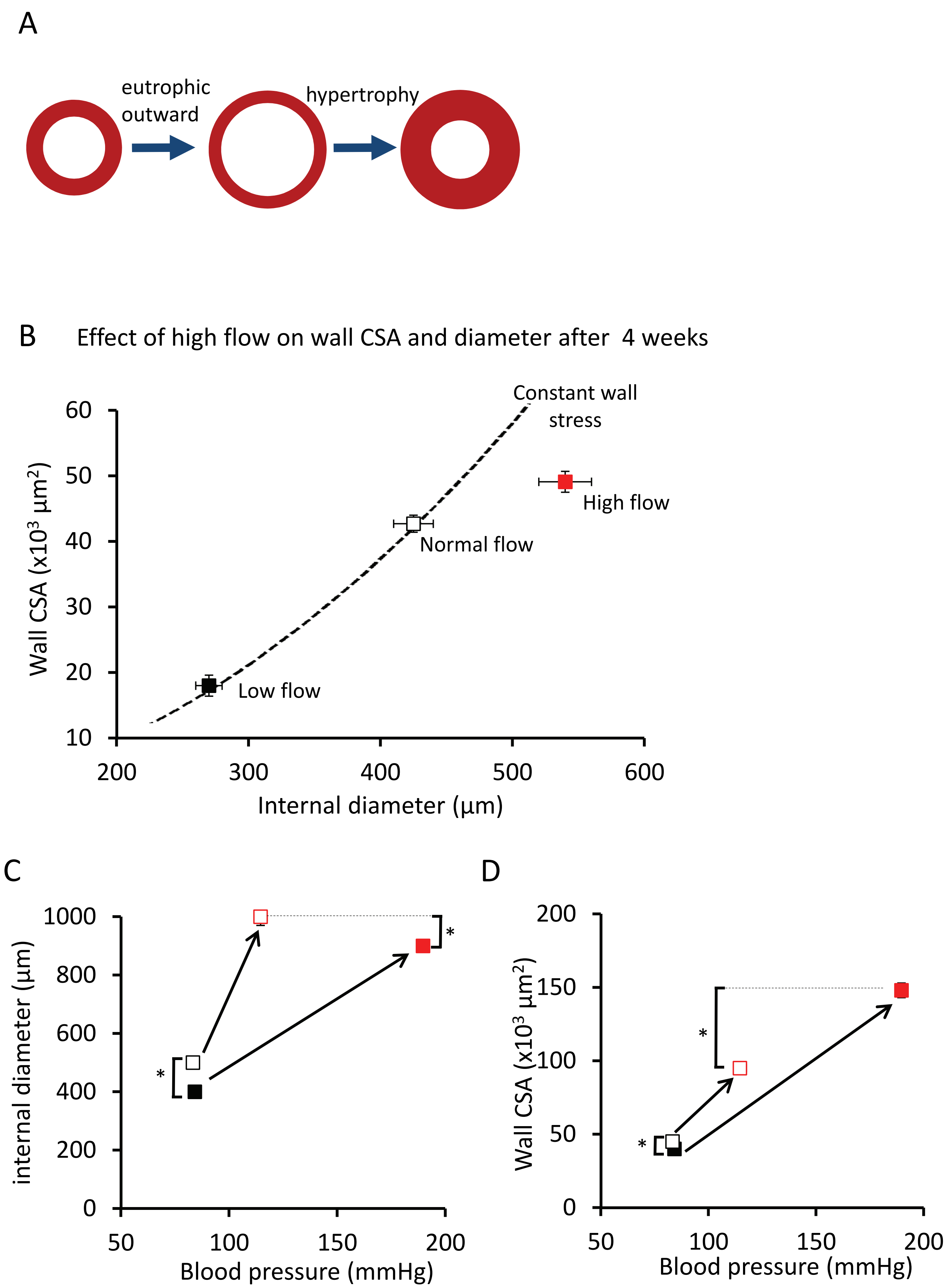
A
eutrophic
outward
hypertrophy
B
Effect of high flow on wall CSA and diameter after 4 weeks
Constant wall stress
High flow
Normal flow
Low flow
Wall CSA (x10³ µm²)
Internal diameter (µm)
60
50
40
30
20
10
200
300
400
500
600
C
internal diameter (µm)
1000
800
600
400
200
0
50
100
150
200
Blood pressure (mmHg)
*
*
D
Wall CSA (x10³ µm²)
200
150
100
50
0
50
100
150
200
Blood pressure (mmHg)
*
*

Fig S5

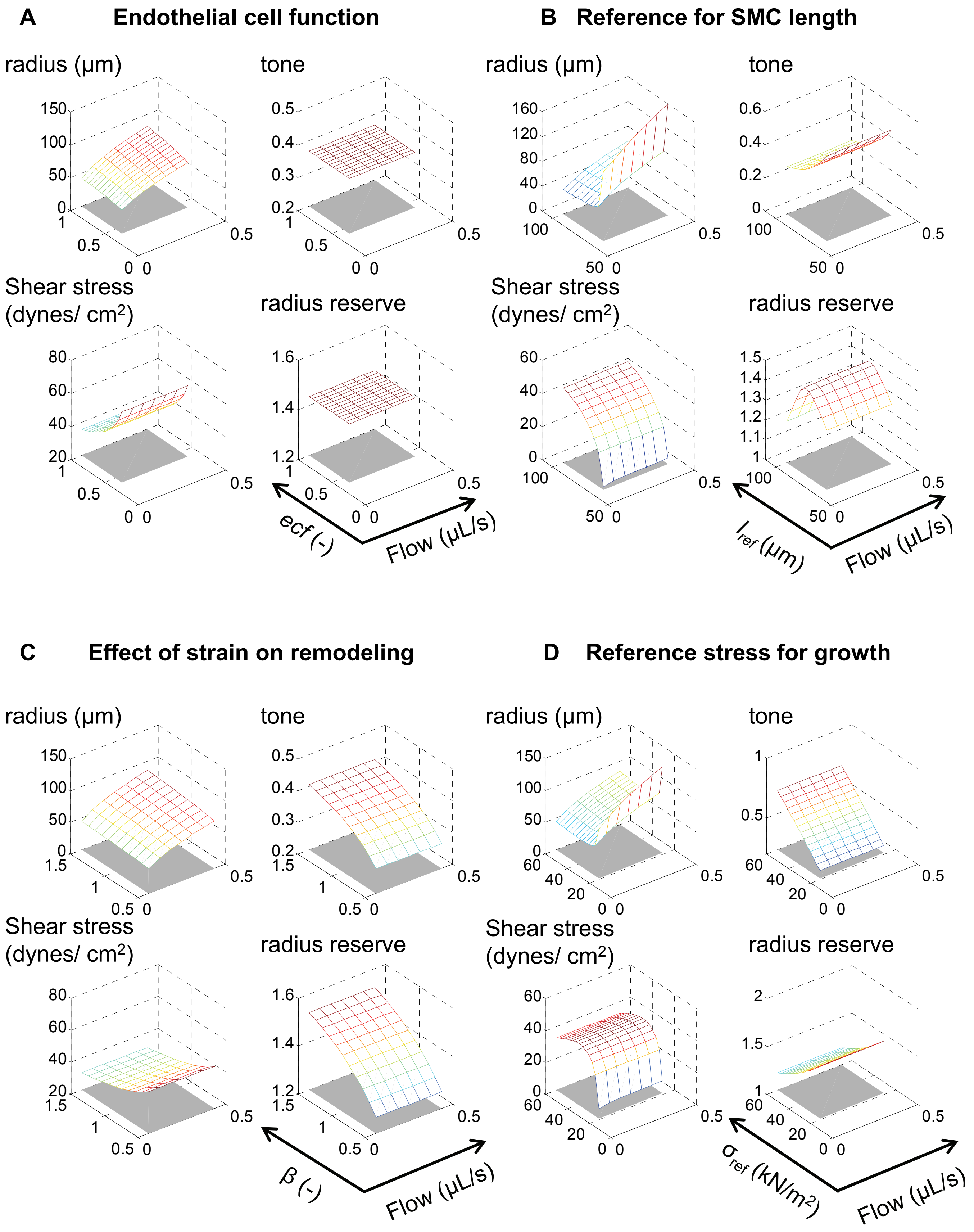
A Endothelial cell function
B Reference for SMC length
C Effect of strain on remodeling
D Reference stress for growth
radius (μm)
tone
Shear stress (dynes/ cm²)
radius reserve
ecf (-)
Flow (μL/s)
l_ref (μm)
β (-)
σ_ref (kN/m²)

Fig S6
